# Leucine Rich Repeat Kinase 2 and Innate Immunity

**DOI:** 10.3389/fnins.2020.00193

**Published:** 2020-03-10

**Authors:** Diba Ahmadi Rastegar, Nicolas Dzamko

**Affiliations:** Brain and Mind Centre, Central Clinical School, University of Sydney, Sydney, NSW, Australia

**Keywords:** LRRK2, Parkinson’s, Crohn’s, inflammation, monocyte, toll-like receptor, inflammasome

## Abstract

For more than a decade, researchers have sought to uncover the biological function of the enigmatic leucine rich repeat kinase 2 (LRRK2) enzyme, a large multi-domain protein with dual GTPase and kinase activities. Originally identified as a familial Parkinson’s disease (PD) risk gene, variations in LRRK2 are also associated with risk of idiopathic PD, inflammatory bowel disease and susceptibility to bacterial infections. LRRK2 is highly expressed in peripheral immune cells and the potential of LRRK2 to regulate immune and inflammatory pathways has emerged as common link across LRRK2-implicated diseases. This review outlines the current genetic and biochemical evidence linking LRRK2 to the regulation of innate immune inflammatory pathways, including the toll-like receptor and inflammasome pathways. Evidence suggests a complex interplay between genetic risk and protective alleles acts to modulate immune outcomes in a manner dependent on the particular pathogen and cell type invaded.

## Introduction

Leucine-rich repeat kinase 2 (LRRK2) first came to prominent attention in 2004, when linkage analysis and positional cloning uncovered *LRRK2* mutations associating with autosomal dominantly inherited Parkinson’s disease (PD) (Paisan-Ruiz et al., 2004; Zimprich et al., 2004). More than fifteen years later, it is now regarded that *LRRK2* pathogenic mutations are the most common cause of dominantly inherited PD. Subsequently, there have been many studies conducted to determine both the physiological and pathophysiological roles of LRRK2.

**FIGURE 1:**
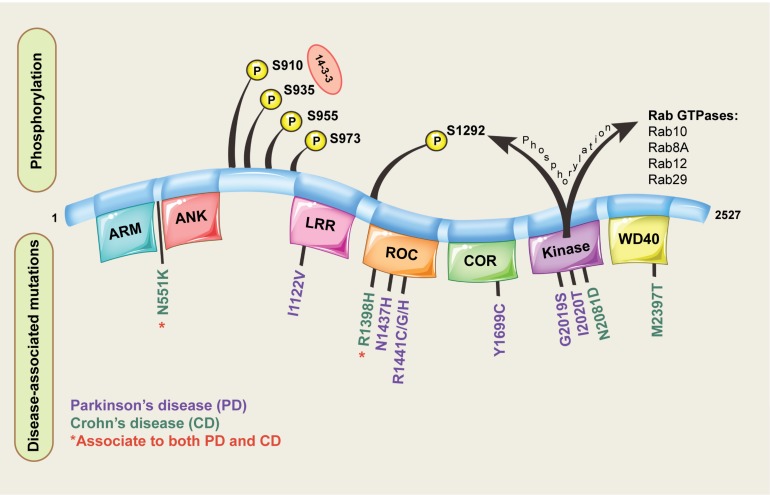
Domain structure of LRRK2. The different domains encoded by the LRRK2 protein are shown, along with pathogenic missense mutations implicated in disease and key phosphorylation residues located on the LRRK2 protein. For the LRRK2 protein domains: ARM = armadillo repeats, ANK = ankyrin repeats, LRR = leucine-rich repeats, ROC = Ras of complex proteins, COR = C-terminal of ROC.

Leucine-rich repeat kinase 2 is located on chromosome 12 and consists of 51 exons encoding a 2527 amino acid protein with a complex domain structure (Figure 1). The encoded protein has several regions involved in protein-protein interactions including a leucine rich repeat domain, an ankyrin repeat domain and a WD40 domain. LRRK2 is also unusual in that it has two domains with catalytic activity; a GTPase domain of the Ras of complex (ROC) protein family, and a kinase domain of the tyrosine kinase like (TKL) family. Both domains seem to be linked, with a complex interplay ultimately regulating catalytic GTPase and kinase activities (Gilsbach and Kortholt, 2014; Liu et al., 2016). The linked activity of the catalytic domains is important, as three missense mutations in the GTPase domain (R1441C, R1441G, R1441H) and two in kinase domain (G2019S and I2020T) are pathogenic for PD, and all lead to an increase in LRRK2 kinase activity (Sheng et al., 2012; Steger et al., 2016). That pathogenic mutations increase LRRK2 kinase activity has provided substantial impetus for the development of LRRK2 kinase inhibitors as potential PD therapeutics (Atashrazm and Dzamko, 2016). Indeed, some studies have suggested efficacy of LRRK2 inhibitors in preclinical studies, and lead compounds are progressing to early stage clinical trials (Alessi and Sammler, 2018; Shihabuddin et al., 2018; Zhao and Dzamko, 2019). However, clinical translation of LRRK2 inhibitors is complicated as the exact biological functions of LRRK2 remain unclear.

One area garnering much attention, is the potential role of LRRK2 in regulating elements of innate immune inflammatory pathways. LRRK2, along with other PD implicated risk proteins, is highly expressed in peripheral immune cells, particularly monocytes (Gardet et al., 2010; Hakimi et al., 2011). In turn, monocytes themselves are increasingly being implicated in PD pathogenesis, largely through potential dysregulation of innate immune inflammatory pathways (Dzamko et al., 2014; Grozdanov et al., 2014; Raj et al., 2014). Indeed, converging evidence suggests a role for familial PD proteins to modulate risk through altered responses to pathogen invasion (Sliter et al., 2018; Matheoud et al., 2019; Shutinoski et al., 2019). The link of LRRK2 to innate immune inflammatory pathways is further strengthened by findings that *LRRK2* polymorphisms also enhance the risk of developing inflammatory bowel disease (Barrett et al., 2008; Franke et al., 2010). Functional studies have also highlighted important roles for LRRK2 in the clearance of bacterial pathogens, such as *Salmonella typhimurium* and *Mycobacteria* (Herbst and Gutierrez, 2019). This review will provide an update on the role of LRRK2 in innate immunity and possible ways in which LRRK2 may contribute to disease pathogenesis.

## LRRK2 is Linked to Diseases with an Innate Immune Component

Originally implicated in PD, subsequent association of *LRRK2* polymorphisms with other diseases has expanded interest to new fields. In particular, there are strong associations between *LRRK2* variants and diseases with inflammatory and/or immune components (Table 1).

**TABLE 1:** Genetic variations in *LRRK2* associated with disease.

Variation	Location	Disease
G2019S	kinase domain	PD
R1441C/G/H	ROC domain	PD
Y1699C	COR domain	PD
I2020T	kinase domain	PD
N2081D	kinase domain	CD
Rs11175593 *LRRK2/MUC19*	non-coding region	CD
Rs11564258 *LRRK2/MUC19*	non-coding region	CD/UC
M2397T	WD40 domain	CD/Leprosy
R1398H	GTPase domain	PD/CD
N551K	In linkage with R1398H	PD/CD
Rs1873613	Promoter	Leprosy
Rs1491938	non-coding region	Leprosy
R1628P	COR domain	Leprosy

*A number of genetic variations in both coding and non-coding regions of the *LRRK2* gene have been associated with Parkinson’s disease (PD), Crohn’s disease (CD), ulcerative colitis (UC) and susceptibility to Leprosy infection.*

## LRRK2 is Genetically Implicated in Disease

Among the pathogenic *LRRK2* mutations linked to PD, the substitution of Gly at amino acid 2019 to Ser (G2019S) is often considered the most common, and is not only found in familial PD, but is also observed in ∼1–5% of sporadic PD cases (Healy et al., 2008). However, the frequency of this mutation varies with ethnic background, and may contribute less to PD in certain European or Asian populations (Shu et al., 2019). The G2019S substitution occurs within the conserved “DFG” motif of the LRRK2 kinase domain, that protects the active site and has a modulatory role in kinase activity. As a result of the substitution, G2019S LRRK2 shows enhanced kinase activity by two to three-fold (West et al., 2005; Jaleel et al., 2007). More than 200 mutations have been reported across the *LRRK2* sequence, with at least a further five kinase activating mutations (R1441C/G/H, Y1699C and I2020T) being confirmed as pathogenic for familial PD (Paisan-Ruiz, 2009). Whilst a number of other non-synonymous *LRRK2* variants associate with both increased or indeed decreased risk of PD including a protective N551K-R1398H-K1423K haplotype (Ross et al., 2011). Finally, genome-wide association studies (GWAS) have consistently identified polymorphisms in the *LRRK2* loci that associate with PD risk in sporadic populations (Satake et al., 2009; Simon-Sanchez et al., 2009; Sharma et al., 2012; Nalls et al., 2014). Thus, the genetic link of LRRK2 to PD is very strong, although understanding exactly how mutations contribute to PD risk is complicated. The penetrance of *LRRK2* mutations is also incomplete (Lee et al., 2017), suggesting that gene-environment interactions likely contribute to individual risk (Pang et al., 2019).

With the proliferation of GWAS studies, interest in LRRK2 was not confined to the PD field for long. Inflammatory bowel disease (IBD) encompasses disorders including Crohn’s disease (CD) and ulcerative colitis (UC) that result from chronic inflammation of intestinal cells due to an abnormal host response to microbiota. The polymorphism rs11175593 located in the loci containing *LRRK2* and *MUC19* was first linked to CD following a meta-analysis of GWAS (Barrett et al., 2008). Further meta-analyses of GWAS data including both CD and UC patients, identified rs11564258 at the same loci, confirming the significant association with candidate genes *LRRK2* and *MUC19* and the risk of IBD (Franke et al., 2010). The rs11564258 polymorphism is only one association out of ∼160, that collectively explain only ∼20% of the variance of IBD (Franke et al., 2010). Nonetheless, in a study using a European population, this SNP had one of the highest risk odds ratios, second only to IL23R for IBD patients compared to controls (Franke et al., 2010). Like PD however, *LRRK2* genetic variation in IBD may display ethnic specificity with common European ancestry *LRRK2* polymorphisms as an example, failing to associate with IBD in an east Asian cohort (Liu et al., 2015). Indeed, an alternate non-synonymous SNP rs3761863, encoding M2397T LRRK2, seems more associated with CD risk in Asian populations (Liu T. C. et al., 2017). With interest in the role of LRRK2 in CD increasing, Hui and colleagues recently performed whole exome sequencing of an Ashkenazi Jewish cohort with CD, leading to the discovery of the non-synonymous variant rs33995883 encoding LRRK2 N2081D (Hui et al., 2018). The N2081D mutation has an odds ratio of 1.3 and is located in the kinase domain of LRRK2, potentially adding to the number of pathogenic kinase activating mutations in this domain. Interestingly, the association signal from previous GWAS identified *LRRK2* SNPs was dependent on the N2081D mutation (Hui et al., 2018). Moreover, the authors also identified a protective haplotype involving the N551K and R1398H LRRK2 variants, previously detected for PD. That both PD and CD share *LRRK2* risk alleles is of interest and leads to questions regarding how *LRRK2* variants may influence progression of one disease or the other. It would also be of interest to determine if *LRRK2* is the sole PD risk gene that overlaps with IBD, or if other familial PD genes confer shared risk.

Leucine-rich repeat kinase 2 has also been genetically linked to Leprosy, a chronic dermato-neurological disorder caused by long-term infection with *Mycobacterium leprae.* Based on the clinical symptomology resulting from bacterial load and individual immune responses, leprosy acts as a spectrum of disease ranging from paucibacillary to multibacillary subtypes (Gaschignard et al., 2016). GWAS analysis of a Han Chinese cohort first suggested an association of *LRRK2* rs1873613 with Leprosy *per se*, and in particular a significant association of *LRRK2* rs1491938 with the multibacillary form of leprosy (Zhang F. R. et al., 2009). Additional studies have supported the association of *LRRK2* variants with Leprosy outcomes, however results are not always consistent across populations or Leprosy subtypes (Wong et al., 2010; Grant et al., 2012; Marcinek et al., 2013; Wang et al., 2015), with analysis presumably complicated by the lower sample sizes available for study of this rarer disease. In ∼30% of patients, Leprosy is associated with acute inflammatory reactions that can lead to debilitating outcomes, in particular pro-inflammatory type-1 reactions (T1R) (Naafs and Van Hees, 2016). Fava and colleagues compared *LRRK2* polymorphisms between Leprosy patient families affected and free from type-1 reactions, and concluded that the majority of GWAS reported *LRRK2* polymorphisms were actually associated with T1R susceptibility within Leprosy, rather than Leprosy susceptibility *per se* (Fava et al., 2016). The largest association with T1R susceptibility was the rs3761863 SNP previously identified for CD, that encodes the LRRK2 M2397T variant (Fava et al., 2016). However, in a replication study, the same authors demonstrate that the main association between LRRK2 and T1R susceptibility, is actually provided by a protective variant (R1628P) enriched in T1R-free subjects (Fava et al., 2019). Thus, as for PD and IBD, the genetics underlying *LRRK2* susceptibility to Leprosy or subsequent complications, likely involves a complex interplay of both risk and protective alleles.

## LRRK2 is Highly Expressed in Innate Immune Cells

**FIGURE 2:**
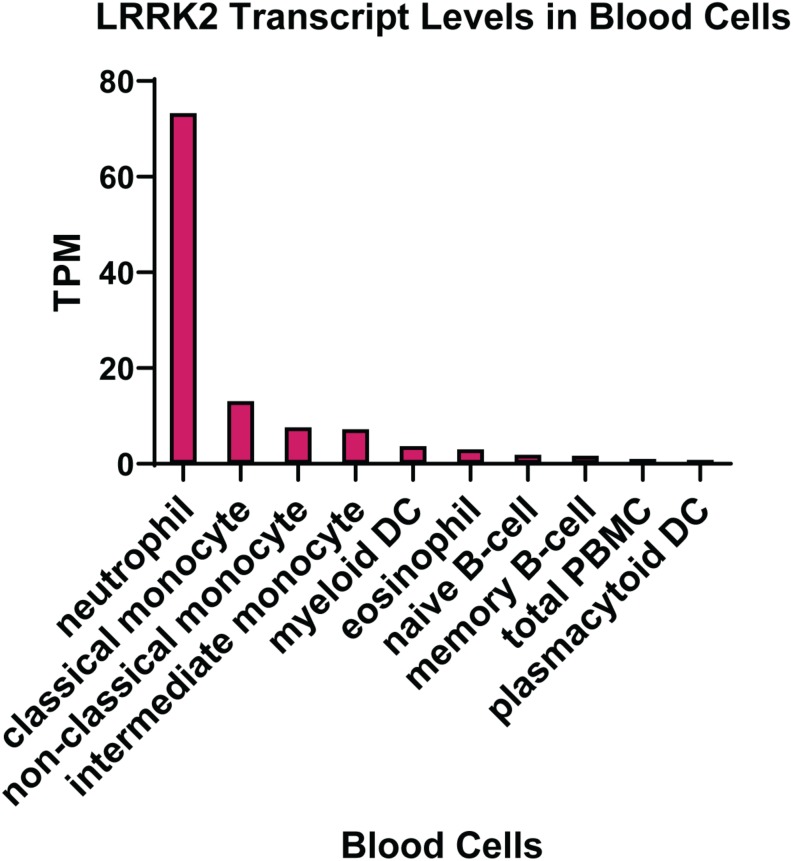
LRRK2 expression in immune cells. Transcript per million (TPM) levels of LRRK2 in different white blood cell immune types. Data was extracted from the human protein atlas (Uhlen et al., 2015) https://www.proteinatlas.org/ENSG00000188906-LRRK2.

The expression of LRRK2 in different tissue types has been extensively studied, with the conclusion that the highest levels are found in peripheral immune cells. In particular, neutrophils and myeloid cells, including monocytes and dendritic cells, express high levels of LRRK2 mRNA (Figure 2). The expression of LRRK2 in monocytes has been assessed at the protein level with the non-classical CD14^+^CD16^+^ pro-inflammatory monocytes expressing LRRK2 the highest (Gardet et al., 2010; Thevenet et al., 2011; Moehle et al., 2015). For lymphoid cells, human CD19^+^ B cells and murine B-2 cells show LRRK2 protein expression (Thevenet et al., 2011), whereas T lymphocytes (CD4^+^, CD3^+^, and CD8^+^) and natural killer cells do not highly express LRRK2 protein, at least under innocuous conditions. That is to say that the expression of LRRK2 protein in immune cells is inducible, particularly by interferon gamma (IFNγ) stimulation, where the *LRRK2* promoter has a conserved binding site for IFN response factors (Gardet et al., 2010). Increased LRRK2 protein has also been observed following stimulation of mouse macrophage cells with the toll-like receptor (TLR) 4 agonist lipopolysaccharide (LPS) (Hakimi et al., 2011). These studies were performed prior to the discovery of LRRK2 substrates so it remains to be determined how LPS and IFNγ impact on LRRK2 activity. Thus, the expression pattern of LRRK2 in immune cell types may be altered under pathophysiological conditions. This is evident from immunophenotyping of PD patient PBMCs, which indicates increased LRRK2 protein in PD patient monocytes (Bliederhaeuser et al., 2016; Cook et al., 2017). Moreover, an increased induction of LRRK2 protein was observed in CD8 + T cells from PD patients following stimulation with IFNγ (Cook et al., 2017). LRRK2 protein was also significantly increased in neutrophils from PD patients (Atashrazm et al., 2019), while LRRK2 mRNA transcription was increased in B cells from patients with systemic lupus erythematosus (SLE) (Zhang et al., 2019), and in macrophages and dendritic cells localized in inflamed intestinal tissue biopsies from CD patients (Gardet et al., 2010).

Leucine-rich repeat kinase 2 is also expressed in the central nervous system (CNS), although the relative expression across different cell types and/or brain regions has been more difficult to ascertain, largely due to specificity issues and reproducibility when using LRRK2 antibodies in fixed brain tissue (Davies et al., 2013). With these caveats in mind, assessment of post-mortem human brain suggests that LRRK2 protein is expressed at low levels in neurons, with the highest expression in astrocytes (Dzamko et al., 2017). Transcriptomic analysis of purified brain cell populations from humans and mice also show robust detection of LRRK2 transcripts in astrocytes and neurons, with substantially lower detection in microglia (Zhang et al., 2014; Booth et al., 2017). Given the high expression in peripheral monocytes, it was generally expected that LRRK2 would also be prevalent in microglia, the resident immune cells of the brain. However, consensus regarding the expression of LRRK2 in microglia is less clear. In initial studies, LRRK2 protein could be detected in murine microglia following acute LPS administration, but not under normal conditions (Moehle et al., 2012). By immunoblotting of primary cultures, low levels of LRRK2 could be detected in microglia from rodents, and this could be further induced with LPS treatment (Moehle et al., 2012). However, more recently and using chronic systemic LPS administration, LRRK2 protein could not be detected and was not upregulated in microglia from transgenic LRRK2 mutation overexpressing mice (Kozina et al., 2018). Studies of post-mortem human brain have also failed to convincingly demonstrate robust LRRK2 expression in microglia (Higashi et al., 2007; Hakimi et al., 2011; Sharma et al., 2011; Dzamko et al., 2017). These studies are additionally complicated by difficulties in accurately separating microglia from high LRRK2 expressing myeloid cells, which are present in blood vessels and may further infiltrate brain tissue under pathological conditions. Thus, if LRRK2 is present in microglia it appears to be at a very low level, at least under normal conditions. A low expression level does not discount biological relevance however, and it is currently unknown whether LRRK2 is upregulated in glia cells in neuroinflammatory disorders.

## LRRK2 is Linked to Innate Immune Signaling Pathways

**FIGURE 3:**
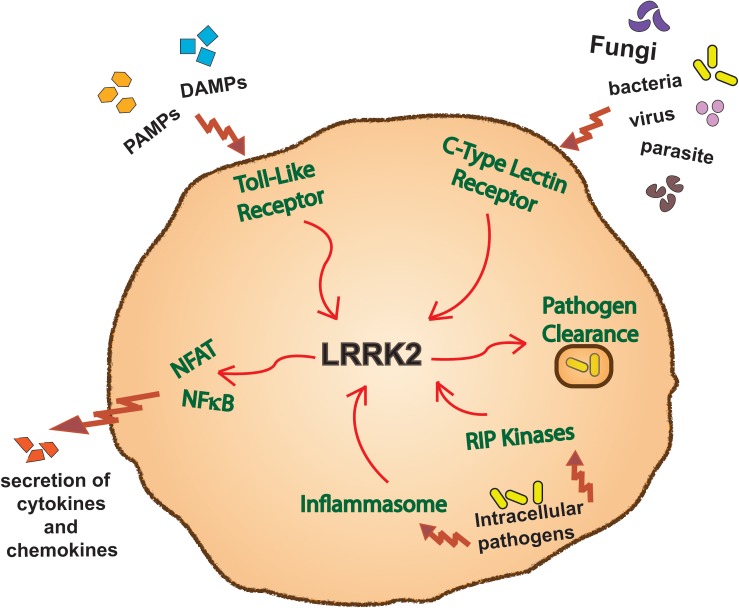
LRRK2 function in immune pathways. Overview of a myeloid cell outlining the innate immune inflammatory pathways in which LRRK2 has been reported to function in.

Both the genetics and expression pattern of LRRK2 have provided impetus for research into specific functions of LRRK2 in innate immune signaling pathways. Although many details remain to be determined, studies on both circulating and infiltrating myeloid cells in mice and humans have already implicated LRRK2 in a number of such pathways (Figure 3).

## LRRK2 and Toll-Like Receptor Signaling

Toll-like receptors (TLRs) are a family of transmembrane proteins that recognize both pathogen-associated molecular patterns (PAMPs) and danger-associated molecular patterns (DAMPs) through binding to their leucine-rich repeat domain (Kawai and Akira, 2011). The classical TLR signal transduction cascade comprises of two different downstream pathways. One, through the myeloid differentiation primary response 88 (MyD88) adaptor protein, increases kinase activity of mitogen activated protein kinase (MAPK) and IκB kinase (IKKα/β) pathways leading to activation of activator protein 1 (AP-1) and nuclear factor kappa B (NFκB) transcription factors and subsequent inflammatory cytokine production. The second, TIR-domain-containing adaptor inducing IFNβ (TRIF)-dependant pathway increases kinase activity of IKKε and tank binding kinase 1 (TBK1) leading to activation of the interferon regulatory factors (IRF3/7) and subsequent production of type 1 IFN. All TLRs signal through the MyD88 protein except TLR3, which recognizes double stranded viral RNA and signals through the TRIF adaptor protein. TLR4, the receptor for LPS, can signal through both MyD88 and TRIF pathways (Dzamko, 2017). Importantly though, these downstream pathways are not mutually exclusive, and crosstalk exists (Clark et al., 2011). Indeed, activation of MyD88-dependent TLRs results in the direct phosphorylation of LRRK2 at Ser910 and Ser935 by IKKε and TBK1 (Dzamko et al., 2012). The Ser910 and Ser935 phosphorylation sites mediate interaction of LRRK2 with 14-3-3 family adaptor proteins, which might be important for the LRRK2 subcellular localization (Nichols et al., 2010). Indeed, activation of TLR4 with LPS causes a redistribution of LRRK2 to membrane structures (Schapansky et al., 2014), and LRRK2 localizes to phagosome structures in monocytes infected with *S. typhimurium* (Gardet et al., 2010). LRRK2 is also required for co-recruitment of Rab proteins to late phagosomes in human IPS-derived macrophages exposed to different TLR2 and TLR4 activating pathogens (Lee et al., 2019). Further details of what occurs downstream of LRRK2 in TLR signaling remain to be elucidated, but phosphorylation of substrate Rab proteins is likely of interest given the published roles they may play in TLR biology (Wang et al., 2010; Luo et al., 2014). A number of studies have also linked LRRK2 to direct regulation of MAPK (White et al., 2007; Gloeckner et al., 2009; Reinhardt et al., 2013) and NFκB (Russo et al., 2015; Lopez De Maturana et al., 2016; Han et al., 2017) signaling pathways, which may have implications for TLR-mediated inflammatory cytokine production. Omics meta-analysis has also linked LRRK2 to dual specificity phosphatases (DUSPs), DUSP1 and DUSP16 in TLR4 signaling, serving as a potential DUSP-mediated hub in this immune modulating pathway (Subbannayya et al., 2019). Thus, biochemical and functional studies clearly link LRRK2 to TLR signaling, with obvious potential implications for LRRK2 function in inflammatory diseases. Moreover, in the context of PD, the pathological α-synuclein protein has been reported to activate TLRs (Beraud and Maguire-Zeiss, 2012) and it will be of interest to determine if TLR activation results in increased LRRK2 activity.

## LRRK2 and C-Type Lectin Signaling

C-type lectin receptors (CLRs) are large superfamily of proteins, often predominantly expressed on myeloid cells, that function as pattern recognition receptors and regulate immunity upon detecting a diverse array of self and non-self ligands (Brown et al., 2018). Like TLR activation, CLR activation also promotes a pro-inflammatory phenotype via activation of NFκB. Using transgenic mice overexpressing LRRK2 it was demonstrated that increased LRRK2 potentiated NFκB-mediated inflammation downstream of the dectin-1 CLR. Activation of dectin-1 with the glucan zymosan resulted in potentiated production of pro-inflammatory cytokines from bone marrow derived dendritic cells of transgenic mice, that was not observed with selective activation of TLR2 (Takagawa et al., 2018). LRRK2 is also reported as a regulator of nuclear factor of activated t cells (NFAT)-dependent cytokine production. Zymosan treatment of LRRK2 knockout bone marrow derived macrophages resulted in higher levels of IL-12 and IL-6 compared to wild type, and again this was not seen with selective activation of TLR2 (Liu et al., 2011). Infection of mouse bone-marrow derived dendritic cells with *Aspergillus fumigatus* also resulted in the downregulation of LRRK2 protein and increased NFAT transcriptional activity (Wong et al., 2018). However, overexpression of LRRK2 has also been demonstrated to increase NFAT transcriptional activity in bone marrow derived dendritic cells (Takagawa et al., 2018), complicating the original interpretation of LRRK2 as a negative regulator of NFAT. Zymosan has also been shown to increase LRRK2 Ser910 andSer935 phosphorylation and LRRK2 localization (Dzamko et al., 2012; Lee et al., 2019), although these effects are potentially mediated via TLR2 rather than dectin-1.

## LRRK2 and Inflammasome Signaling

Inflammasomes are multiprotein signaling complexes that also play a key role in pathogen recognition and innate immunity that have predominantly been studied in monocytes, macrophages and microglia. Canonical inflammasome activation causes cleavage of pro-caspase 1, which in turn cleaves precursor pro-IL-1β and pro-IL-18, leading to release of the respective biologically active cytokines (Latz et al., 2013). Inflammasome complexes are grouped based on the different pattern recognition receptors (PRRs) that act as the sensor molecules (Guo et al., 2015). In particular, LRRK2 has been reported as an essential component for the complete activation of NLR family CARD domain-containing protein 4 (NLRC4) inflammasome in mice infected with *S. typhimurium* (Liu W. et al., 2017). Moreover, LRRK2 was shown to phosphorylate NLRC4 at Ser533 (Liu W. et al., 2017), a key residue for inflammasome formation (Qu et al., 2012). The interaction between LRRK2 and NLRC4 was mediated via interaction of the WD40 domain of LRRK2 and the LRR domain of NLRC4 (Liu W. et al., 2017). These findings were specific for NLRC4, as LRRK2 did not modulate the function of the NLRP3 inflammasome. Intriguingly, inhibition of the NLRP3 inflammasome, rather than NLRP4 have had recent success in treating rodent models of Parkinson’s disease (Gordon et al., 2018). Thus, further work to understand the function of LRRK2 in broader inflammasome activation and the consequence for LRRK2-implicated inflammatory diseases will be of interest.

## LRRK2 and Receptor Interacting Protein Kinase Signaling

LRRK2 is located in the seven-member ser/threonine receptor interacting protein kinase (RIPK) branch of the human kinome, with LRRK2 alternately classified as RIPK7 (Dzamko and Halliday, 2012). Most RIPK family members have known roles in immunity and the regulation of cell death pathways (Zhang et al., 2010), and it has been regarded that LRRK2 may have common conserved biological functions. The best studied RIPK family member is RIPK2, which is recruited to the intracellular pathogen sensing nucleotide-binding oligomerization domain-containing protein 2 (NOD2) receptor upon its association with the bacterial peptidoglycan muramyl dipeptide. This binding results in an NFκB-mediated inflammatory cytokine response (Inohara et al., 2003). Like LRRK2, mutations in RIPK2 and NOD2 are also associated with increased susceptibility to Crohn’s disease (Hugot et al., 2001; Ogura et al., 2001; Umeno et al., 2011). In overexpression experiments, LRRK2 has been shown to physically interact with RIPK2, and LRRK2 kinase activity promoted phosphorylation of RIPK2 on Ser176 (Yan and Liu, 2017), a reported RIPK2 regulatory autophosphorylation site (Dorsch et al., 2006). LRRK2 also interacts with RIPK1, a signal transducer downstream of death receptors. In particular, it has been demonstrated that LRRK2 co-immunoprecipitates with the death domain containing proteins RIPK1, FADD and TRADD (Ho et al., 2009). The interaction between LRRK2 and RIPK1 was increased following TNFα-mediated induction of RIPK1-dependent apoptosis (Amin et al., 2018). Moreover, the use of LRRK2 siRNA and LRRK2 KO MEFS demonstrated a requirement for LRRK2 in the formation of a distinct insoluble and ubiquitinated RIPK1 intermediate, that specifically promoted RIPK1-dependent apoptosis (Amin et al., 2018). This concept is analogous, but distinct, from RIPK1 modulating RIPK3 activity to promote TNFα-mediated necroptosis (Zhang D. W. et al., 2009), and is of interest as RIPK1 is also a potential therapeutic target for inflammatory diseases including PD (Yuan et al., 2019).

## LRRK2 Biological Function in Innate Immunity

**FIGURE 4:**
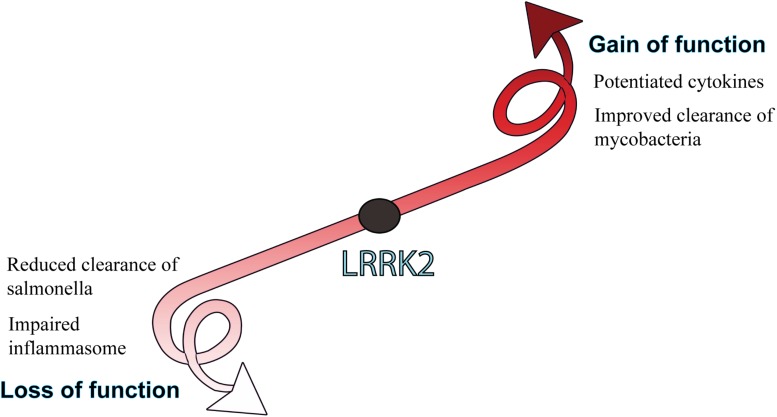
Biological consequences of LRRK2 function. LRRK2 mutations or increased levels that lead to a gain of function are generally associated with potentiated immune responses, whereas LRRK2 loss of function is more associated with an impaired host-immune response to pathogens. How gain or loss of LRRK2 function influences immune outcomes is largely dependent on the pathogen and cell type being studied.

Given the high expression in myeloid cells and links to innate immune signaling pathways, immune studies of LRRK2 gain and loss of function have particularly focused on the host response to pathogens (Figure 4).

## LRRK2 and Inflammatory Cytokines

Although initial studies using LRRK2 KO mouse macrophages showed no effect on TLR-mediated inflammatory cytokine secretion (Hakimi et al., 2011; Dzamko et al., 2012), the extent to which LRRK2, and/or activity modulating *LRRK2* variants may contribute to inflammatory cytokine levels continues to be investigated. In people, higher levels of serum IL-1β, TNFα, IL-6, IL-10, and MCP1 were observed in only a subgroup of asymptomatic carriers of the LRRK2 G2019S mutation (Dzamko et al., 2016). Once PD is manifesting, patients with the LRRK2 G2019S mutation do not appear to have higher inflammatory cytokine levels than idiopathic patients (Dzamko et al., 2016; Ahmadi Rastegar et al., 2019). This, along with the fact that LRRK2 G2019S is not fully penetrant for disease, may suggest that *LRRK2* mutations themselves do not drive inflammatory pathway activation, but rather may modulate responses to inflammatory pathway activation. Indeed, some evidence is suggestive of a role for LRRK2 to potentiate inflammatory cytokine responses downstream of pattern recognition receptors. In particular, transgenic mice overexpressing the R1441G mutation show a marked increase in peripheral levels of cytokines IL-6, IFNγ, IL-10, CCL5, M-CSF, and G-CSF following systemic LPS administration (Kozina et al., 2018). A similar result of potentiated inflammatory cytokines was obtained with LPS treated primary cells from R1441G transgenic mice (Gillardon et al., 2012). However, potentiated cytokine profiles were not observed in studies employing LPS treated G2019S transgenic mice (Moehle et al., 2015; Litteljohn et al., 2018), or G2019S mouse macrophages infected with *S. typhimurium* (Shutinoski et al., 2019). Thus, it seems that context is important for LRRK2 immunological responses and perhaps unsurprisingly, responses may differ between mutations, species and nature of the stimuli. Outside of TLR activation, LRRK2 has also been implicated in modulating the inflammatory cytokine levels in response to CLR and inflammasome agonists. Stimulation of transgenic LRRK2 overexpressing bone marrow derived dendritic cells with dectin agonists, ZymD, heat killed *S. cerevisiae* and heat killed *C. albicans* resulted in increased production of TNFα, IL-23, and IL-2 (Takagawa et al., 2018). Bone marrow derived macrophages from G2019S LRRK2 transgenic mice also showed increased production of IL-1β following activation of the NLRC4 inflammasome with *S. typhimurium* (Liu W. et al., 2017), and both the G2019S and R1441C LRRK2 mutations increased NFκB activation and IL-8 production following stimulation of transfected HEK293 cells with IL-1β (Han et al., 2017). Thus, although still inconclusive, at least some collective evidence to date points toward a potentiated response to inflammatory stimuli when LRRK2 is upregulated and/or activated.

## LRRK2 and Pathogen Clearance

As well as the inflammatory response to pathogens, there has also been informative research conducted into how LRRK2 function may modulate the clearance of intracellular pathogens. Initial studies using siRNA knockdown of LRRK2 in RAW macrophage cells demonstrated that reduced LRRK2 protein was associated with impaired clearance of *S. typhimurium*, likely due to an impaired antibacterial response to generate reactive oxygen species (Gardet et al., 2010). Impaired clearance of *S. typhimurium* was also observed *in vivo* using LRRK2 knockout mice, which were more markedly more susceptible to infection and unable to mount a sufficient inflammasome response (Liu W. et al., 2017). Intriguingly, mice with the G2019S LRRK2 mutation showed improved bacterial control of *S. typhimurium* (Shutinoski et al., 2019), adding to suggestions that some LRRK2 mutations may constitute an evolutionary advantage against infection (Herbst and Gutierrez, 2019). Indeed, LRRK2 kinase activity has also been associated with improved clearance of *M. tuberculosis* (Hartlova et al., 2018) and protective alleles identified that improve clearance of *M. leprae* (Fava et al., 2019). However, context again appears to be important with LRRK2 knockout mice showing increased susceptibility to *L. monocytogene*s (Zhang et al., 2015). That the host response to different pathogens that infect different cell types in different manners can be either positively or negatively impacted by LRRK2 function certainly complicates research in this area. In terms of mechanistic insight, studies to date suggest that LRRK2 may modulate the uptake, trafficking and lysosomal degradation of pathogens, impact upon mitochondrial function thereby reducing the capacity of reactive oxygen species to target pathogens and/or modulate inflammatory responses through the regulation of immune signaling pathways such as the inflammasome (Gardet et al., 2010; Zhang et al., 2015; Liu W. et al., 2017; Hartlova et al., 2018; Herbst and Gutierrez, 2019; Lee et al., 2019).

## LRRK2 as a Potential Therapeutic Target for Inflammatory Disease

Since the original biochemical discoveries that the LRRK2 G2019S PD pathogenic mutation increased kinase activity, there has been a sustained effort to develop clinically applicable small molecule LRRK2 kinase inhibitors. The development of LRRK2 kinase inhibitors has indeed progressed remarkably, and after a decade of research the DNL201 LRRK2 kinase inhibitor from Denali Therapeutics recently entered phase 1a clinical trials in healthy volunteers. Although this was a small trial and potential long-term side effects are unknown, primary outcomes were promising in showing that acute LRRK2 inhibition is tolerated (Zhao and Dzamko, 2019). DNL201 has now entered a phase 1b study employing PD patients with mild to moderate disease, with and without LRRK2 mutations. A second LRRK2 inhibitor from Denali Therapeutics, DNL-15 is also being tested in a phase 1 study of healthy volunteers and outcomes from these trials are awaited with anticipation. Despite the impressive advances in regard to LRRK2 kinase inhibitors however, the exact LRRK2 biological functions and thus consequences of its pharmacological inhibition are not clear, particularly over a long term. In regard to the immune system, anti-inflammatory properties of LRRK2 inhibitors may contribute to a disease modifying mechanism for PD, potentially via down regulation of microglia or modulating peripheral immunity. With increasing evidence that interplay between genetics and peripheral immunity influences PD risk, there may also be opportunities for earlier intervention in LRRK2 mutation risk carriers with higher levels of inflammation (Dzamko et al., 2016). Moreover, there is also emerging evidence that targeting LRRK2 in IBD patients may also have merit for modulating future PD risk (Rolli-Derkinderen et al., 2019). A key to understanding the therapeutic potential for targeting LRRK2 in the immune system for multifactorial diseases such as PD and IBD however, is likely to require a greater understanding of what initiates inflammation in the first place. As outlined above, the host response to some pathogens may actually be impaired by inhibiting LRRK2 with the potential to exacerbate disease. Model pathogens such as *S. typhimurium* or *M. leprae* are certainly of interest for understanding LRRK2 function in a defined context, but from an epidemiological viewpoint these would seem unlikely major initiators of PD or IBD. This in turn raises the question of how LRRK2 may modulate the response to more ubiquitous pathogens implicated in PD and IBD, such as gut microbiota (Houser and Tansey, 2017; Ni et al., 2017), periodontal microbes (Vavricka et al., 2013; Chen et al., 2017) or influenza virus (Sadasivan et al., 2017). This is likely an interesting area of future research.

Finally, it is noteworthy that not all therapeutic approaches targeting LRRK2 are small molecule kinase inhibitors. For example, anti-sense oligonucleotides have demonstrated efficacy via an ability to reduce LRRK2 levels in the brain of pre-clinical models (Zhao et al., 2017), and a phase 1 trial testing the safety and tolerability of a LRRK2 ASO therapy (BIIB094) is currently being conducted by Biogen and Ionis (NCT03976349). In this trial, the ASO will be administered intrathecally and avoid interfering with peripheral LRRK2. Indeed, there may be merit to designing unique approaches to target specific mutant forms of LRRK2 and/or target therapies to distinct tissues/cell populations. To facilitate such approaches though, a greater understanding of the role of LRRK2 in the immune system and how the enzyme modulates risk across inflammatory diseases still remains to be determined. Meanwhile, the outcomes of current LRRK2 therapeutic trials will be watched with great interest.

## Author Contributions

ND and DA wrote the manuscript and prepared the figures.

## Conflict of Interest

ND has previously received travel support from Denali Therapeutics and Neuropore Therapies, and consultancy funds from Celgene. The remaining author declares that the research was conducted in the absence of any commercial or financial relationships that could be construed as a potential conflict of interest.
